# 

**Published:** 1908-06

**Authors:** 


JUNE, 1908.

*Vol.* XXVI. *No.* 100.

THE

### BRISTOL MEDICO-CHIRURGICAL JOURNAL

*PUBLISHED UNDER THE AUSPICES OF*

*THE BRISTOL MEDICO-CHIRURGICAL SOCIETY.*

*Editor:* R. SHINGLETON SMITH, M.D., B.Sc.

WITH WHOM ARE ASSOCIATED

J. MICHELL CLARKE, M.A., M.D.,

J. LACY FIRTH, M.S., M.D., JAMES SWAIN, M.S., M.D.

P. WATSON WILLIAMS, M.D., *Assistant Editor.*

*Editorial Secretary:* JAMES TAYLOR.

LONDON: J. & A. CHURCHILL, 7 GREAT MARLBOROUGH STREET.

LIBRARY OF CONGRESS
488150
SMITHSONIAN DEPOSIT.

*Price One Shilling and Sixpence.*

